# Carbon (δ^13^C) and Nitrogen (δ^15^N) Stable Isotope Composition Provide New Insights into Phenotypic Plasticity in Broad Leaf Weed *Rumex acetosa* under Allelochemical Stress

**DOI:** 10.3390/molecules23102449

**Published:** 2018-09-25

**Authors:** M. Iftikhar Hussain, Manuel J. Reigosa, Adele Muscolo

**Affiliations:** 1 Department of Plant Biology and Soil Science, University of Vigo, Campus Lagoas-Marcosende, E-36310 Vigo, Spain; mreigosa@uvigo.es; 2 Research Institute of Sciences and Engineering (RISE), University of Sharjah, P.O. Box 27272 Sharjah, UAE; 3 Department of Agriculture, Mediterranean University, Feo di Vito, 89122 Reggio Calabria, Italy; amuscolo@unirc.it

**Keywords:** phenolic compounds, physiological growth, natural herbicide, shoot growth, root growth, phytotoxicity, δ^13^C, δ^15^N, *Rumex acetosa* L.

## Abstract

Phenolic compounds, hydroquinone and cinnamic acid derivatives have been identified as major allelochemicals with known phytotoxicity from allelopathic plant *Acacia melanoxylon* R. Br. Several phenolic compounds such as ferulic acid (FA), *p*-hydroxybenzoic acid (pHBA) and flavonoid (rutin, quercetin) constituents occur in the phyllodes and flowers of *A. melanoxylon* and have demonstrated inhibitory effects on germination and physiological characteristics of lettuce and perennial grasses. However, to date, little is known about the mechanisms of action of these secondary metabolites in broad-leaved weeds at ecophysiological level. The objective of this study was to determine the response of *Rumex acetosa* carbon isotope composition and other physiological parameters to the interaction of plant secondary metabolites (PSM) (FA and pHBA) stress and the usefulness of carbon isotope discrimination (Δ^13^C) as indicative of the functional performance of intrinsic water use efficiency (iWUE) at level of plant leaf. *R. acetosa* plant were grown under greenhouse condition and subjected to PSM stress (0, 0.1, 0.5, 1.0, and 1.5 mM) for six days. Here, we show that FA and pHBA are potent inhibitors of Δ^13^C that varied from 21.0‰ to 22.9‰. Higher pHBA and FA supply enhanced/retard the N_leaf_ and increased the C_leaf_ while ratio of intercellular CO_2_ concentration from leaf to air (*C*i/*C*a) was significantly decreased as compared to control. Leaf water content and leaf osmotic potential were decreased following treatment with both PSM. The *C*i/*C*a decreased rapidly with higher concentration of FA and pHBA. However, iWUE increased at all allelochemical concentrations. At the whole plant level, both PSM showed pronounced growth-inhibitory effects on PBM and C and N concentration, root fresh/dry weight, leaf fresh/dry weight, and root, shoot length of C_3_ broad leaf weed *R. acetosa*. Carbon isotope discrimination (Δ) was correlated with the dry matter to transpiration ratio (transpiration efficiency) in this C_3_ species, but its heritability and relationship to *R. acetosa* growth are less clear. Our FA and pHBA compounds are the potent and selective carbon isotope composition (δ^13^C) inhibitors known to date. These results confirm the phytotoxicity of FA and pHBA on *R. acetosa* seedlings, the reduction of relative water content and the induction of carbon isotope discrimination (Δ) with lower plant biomass.

## 1. Introduction

Herbicide resistance in weeds is now a lethal cascade that is decreasing the crop yield and profit of the farmer worldwide [1,2]. In the USA alone, the annual cost of crop losses due to weeds is greater than $26 billion [3]. In the UK, herbicide resistance in blackgrass (*Alopecurus myosuroides*) has destroyed thousands of farms that further exacerbated the wheat production (approximately £0.5 billion losses) [4]. The evolutionary adaptation is the major source of resistance in the weeds against the herbicides [5]. Natural products from living organisms, plants, fungi and bacteria are a huge source of environmentally friendly “bioherbicides” that can overcome the herbicide resistance problem [6,7]. However, the bioherbicides or secondary metabolites might interfere with the growth, physiological and biochemical process of target plants, but their mode of action has rarely been studied [8]. Some secondary metabolites such as grandiflorone and flavesone have shown significant phytotoxicity and their mechanism of action was inhibition through hydroxyphenyl pyruvate dioxygenase enzyme [9]. Some researchers reported the inhibition of germination, plant growth and physiological processes of crop plants, weeds and vegetables by phenolic compounds. The citral, momilactones, and sorgoleone are the potential source of new compounds that can serve as lead molecules for herbicide discovery program [10].

The chemical structure of allelochemicals are very complex and several modes of action of their phytotoxicity have been elucidated. The agro-physiological attributes and biomass of soybean was significantly reduced by *p*-coumaric and *p*-hydroxybenzoic acids (0.5–1.0 mM) [11]. The pHBA has been reported to release into soil by root exudates, leaf leachates and decomposed tissues of wheat (*Triticum aestivum* L.) [12]. The wild oat (*Avena fatua*) release pHBA via root exudates that ultimately affect the growth, development and biomass of surrounding plants such as wheat [13]. In this study, based on their chemical and structural similarity with the previously introduced C_3_-selective Light Harvesting Complex (LHC II) and carbon isotope composition retarders [14], we identified phenolic compounds as suitable selective inhibitors for C_3_ photosynthetic apparatus (PSII photochemistry).

Previously, we found that the *A. melanoxylon* flower aqueous extract (100%, 75%, and 50%) reduced seedling growth and germination of *D. glomerata*, *R. acetosa, L. perenne*, and *L. sativa*. [15]. Regarding germination inhibition, the mean LC_50_ value of the *A. melanoxylon* flower and phyllodes extracts was 43% and 41% (*L. perenne*), 40% and 38%, in *R. acetosa*, and 53% and 41%, respectively, in *L. sativa* [15]. The fractions obtained were evaluated for identification of the allelochemicals. The most important secondary metabolites were identified as phenolic compounds (ferulic acid, *p*-hydroxybenzoic acid, cinnamic acid, *p*-coumaric acid, gallic acid, protocatequic acid, vanillic acid, and syringic acid) and several flavonoids [16]. The phytotoxicity profiles of some of these selected compounds were assayed on *Lactuca sativa*, *Lolium perenne* and *Dactylis glomerata*, with high bioactivities observed mainly with ferulic acid, *p*-hydroxybenzoic acid, and cinnamic acid [16]. The results summarized here demonstrate that the phytotoxicity of the extracts and the pure compounds showed the capacity of these secondary metabolites for future use in the biological weed control programs. Biochemical and physiological characteristics of these metabolites indicate that the compounds identified from the plant extracts might be responsible for inhibition of germination, seedling growth and interaction of seedling with other plants in the natural environment [17].

Reliable applications of the non-invasive technique of isotope signatures in broad leaf weed, *R. acetosa* for developing herbicide-resistance model and impact of natural compounds in interfering the physiological features required the understanding of the detailed mechanisms of carbon and nitrogen isotope discrimination in leaf organic matter of this plant. Furthermore, natural products from plants offer a broad array of molecules with great diversity in their structure, biological activity and toxicology that can be used for managing weeds [18]. Allelochemicals of plant origin presents alternate option for weed management but it is necessary to understand their mechanism of action. However, only few studies have recently started examining the role of allelochemicals, elucidating the variation in δ^13^C and δ^15^N in the leaf organic matter of glass-house grown vegetables and perennial grasses under controlled conditions. The objectives of the present work were to determine the primary target of action site within the intercellular CO_2_ concentration from leaf to air (*C*i/*C*a) and their interference with relative water content (RWC) and photosynthetic carbon isotope discrimination in *R. acetosa*. Therefore, in the present work, the inhibitory impact of both phenolic compounds (FA and pHBA) was assayed on the metabolism of adult plants of broadleaved weed, *R. acetosa* using isotope ratio mass spectrometer, to establish the primary affected organ and understand the lethal impact of these molecules. The effects of FA and pHBA on RWC (relative water content), δ^13^C (composition of carbon isotope ratios), Δ^13^C (carbon isotope discrimination), and *C*i/*C*a (ratio of intercellular CO_2_ concentration from leaf to air) on Δ were assessed. Specifically, the following three questions were addressed: How do different concentrations of FA and pHBA affect Δ, N, C_leaf_, RWC and LOP? How do both secondary metabolites affect the N_leaf_, iWUE and *C*i/*C*a? Which is the major factor (FA or *p*HBA) affecting Δ, RWC or *C*i/*C*a?

## 2. Material and Methods

### 2.1. Plant Growth Conditions and Treatments

The *R. acetosa* L. (cv. Belleville) seeds were surface sterilized with NaCIO_3_ (0.5%), and then rinsed with distilled water. Seeds were sown individually in plastic trays (32 cm × 20 cm × 6 cm) filled with 5 cm deep layer of perlite (500 g/tray). The seedlings were grown with 10% Hoagland solution at temperature 28/20 °C (day/night), photoperiod 9/15 h (light/dark) and relative humidity of 80%. The other growth conditions were the same as reported previously [6]. The treatment solution of FA and pHBA was prepared (concentrations: 1.5, 1.0, 0.5, and 0.1 mM) from the stock solution (3 mM). The treatments (100 mL/pot) were applied on alternative days for six days. The experiment was laid out in Randomized Complete Block Design (RCBD) with three replications of each treatment and a control.

### 2.2. Carbon Isotope Discrimination and Mass Spectrometry Analysis

The plant leaf samples collected from each treatment and control were dried and ground into fine powder. The encapsulated leaf samples (1700–2100 µg) were subject to isotope analysis using Isotope Ratio Mass Spectrometer (Finnegan: Thermo Fisher Scientific, model MAT-253, Swerte, Germany) at the Stable Isotope facility, University of Vigo, Spain. The isotopic composition was reported as δ^13^C (‰) having Vienna Pee Dee Belemnite (V-PDB) as international standard (R_standard_) and calculated according to the methodology [19,20] by using Equation (1):

δ (‰) = [(R_sample_/R_standard_) − 1)] × 1000 (1)

where R_sample_ is the ratio of ^13^C/^12^C or ^15^N/^14^N, and R_standard_ were the standards used. Atmospheric N_2_ was the standard for nitrogen while Vienna PeeDee Belemnite (VPDB) was the standard for carbon. The accuracy and reproducibility of the measurements of δ^13^C and δ^15^N were checked with an internal reference material (NBS 18 and IAEA-C6 for C), and (IAEA-310A and IAEA- N1 for N), and acetanilide for C/N % ratios, respectively.

The ^13^C discrimination (Δ^13^C) was calculated from the measured values of δ^13^C_sample_ using Equation (2):

Δ^13^C (‰) = [(δ^13^C_air_ − δ^13^C_sample_)/ (1 + δ^13^C_sample_)] × 1000 (2)

where δ^13^C_air_ and δ^13^C_sample_ are the carbon isotope compositions of air and plant samples, respectively. δ^13^C_air_ was considered at −8.15‰, as reported in CDIAC [21]. The intrinsic water use efficiency was calculated using the procedure reported by Robertson et al. [22] and Hussain and Reigosa [14].

### 2.3. Relative Water Content and Leaf Osmotic Potential

The leaf fresh weight (W_f_), saturated (W_t_) and dry (W_d_) of *R. acetosa* were obtained through standard protocol. The osmotic potentials of all treated and control samples were determined through Automatic Cryoscopic Osmometer (Osmomat–030, GmbH, Gonatec, Berlin, Germany), as documented previously [14].

### 2.4. Harvesting and Plant Growth Bioassays

The agro-morphological characteristics (shoot, root length, fresh/dry weight of leaf and root) were measured according to standard procedure as reported previously [14].

### 2.5. Statistical Analysis

One-way ANOVA followed by the Dunnett test (at 0.05 probability) was used to evaluate the effects of FA and pHBA addition on the response variables (RWC, LOP, *C*i/*C*a, iWUE, N_leaf_, and Δ) under the glass house conditions. Data were analyzed to explain the effects of FA and pHBA on RWC, LOP, *C*i/*C*a, iWUE, N_leaf_, and determine the extent to which Δ was influenced by iWUE and *C*i/*C*a. All procedures were carried out in SPSS Version 19.0 (SPSS Inc., Chicago, IL, USA).

## 3. Results

### 3.1. Effects of Phenolic Acids on Photosynthetic Carbon Isotope Discrimination

**Figure 1:**
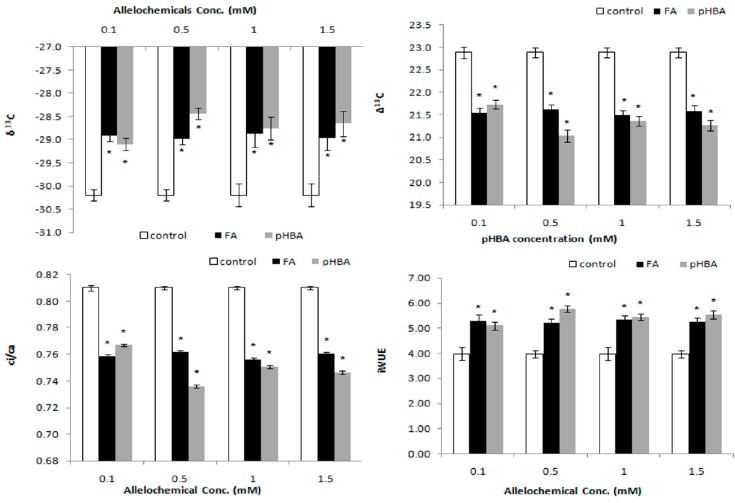
Changes following one-week exposure to ferulic acid (FA) and *p*-hydroxybenzoic acid (pHBA) at 0.1, 0.5, 1.0, 1.5 mM concentrations in: carbon isotope composition (δ^13^C) (**A**); carbon isotopes discrimination (Δ^13^C) (**B**); ratio of CO_2_ from leaf to air (*C*i/*C*a) (**C**); and intrinsic water use efficiency (iWUE) in leaves of *Rumex acetosa* (**D**). * Asterisk indicates significant differences at *p* ≤ 0.05 with respect to control.

The values of Δ^13^C varied from 21.0‰ to 22.9‰. Both FA and pHBA supply showed significant effect on Δ^13^C at *p* = 0.05 (Figure 1). At higher pHBA and FA supply, the *C*i/*C*a was significantly decreased as compared to control. Carbon isotope discrimination (Δ) usually correlates with the dry matter to transpiration ratio (transpiration efficiency) in C_3_ species, but its heritability and relationship to *R. acetosa* growth are not clear. Carbon isotope composition ratio (δ^13^C) was less negative (−28.4) than the control (−30.2) in *R. acetosa* following treatment at highest level of both secondary metabolites (Figure 1). 

The effects of FA and pHBA on δ^13^C and *C*i/*C*a, varied at different levels of treatment (Figure 1). Under low allelochemical concentrations, higher N availability might stimulate CO_2_ carboxylation in RuBisCO. *C*i/*C*a decreased rapidly with higher concentration of FA and pHBA. However, iWUE increased all allelochemical concentrations. Compared to no allelochemical supply, iWUE was significantly higher at all allelochemical concentrations. The ratio of intercellular to ambient CO_2_ concentration in *R. acetosa* was decreased following treatment of both secondary metabolites and this effect was lethal at 0.5, 1.0 and 1.5 mM level (Figure 1).

### 3.2. Carbon and Nitrogen Concentrations and Δ^15^n Isotope Composition 

Increasing FA and pHBA concentration from 0.1 to 1.5 mM produced significant 9.3% and 9.76% declines in C_leaf_, respectively (Table 1). At lower FA concentration (0.1 mM), the N_leaf_ levels were maintained at approximately 1.90% that was at par with control. However, at higher concentration (1.5 mM) of FA, there was a 25.38% reduction in N_leaf_ as compared to control. In contrast, pHBA showed a significant decline of 28.93% in N_leaf_ level as compared to control (Table 1; *p* ≤ 0.05). Increasing the allelochemical concentration from 0.1 to 1.5 mM, FA treatment (1.5 mM) caused 28.29% decrease in leaf nitrogen isotope composition (δ^15^N) in *R. acetosa* while 33.82% reduction in δ^15^N was obtained following treatment at 1.5 mM pHBA (Table 1; *p* ≤ 0.05). Significant differences between the two allelochemicals (FA and pHBA) were observed at 1.0 and 1.5 mM (*p* < 0.05). It appears that both phenolic compounds adopt a strategy to decrease the C_leaf_, N_leaf_ and leaf δ^15^N in *R. acetosa*. It was observed that, once the allelochemical threshold level is exceeded, the physiological mechanism might break down as C_leaf_ and N_leaf_ levels begin to decrease abruptly. The relationship between tissue C_leaf_ and N_leaf_ can be revealed by plotting C/N ratio as a function of external abiotic stress, such as allelochemicals (Table 1). Both FA and pHBA maintained a significant impact on C/N ratio that was in the range of 23–31 with increasing allelochemical concentrations from 0.1 to 1.5 mM. 

### 3.3. Elucidation of Inhibitory Effects of Phenolic Acids on Leaf Water Relations

**Figure 2:**
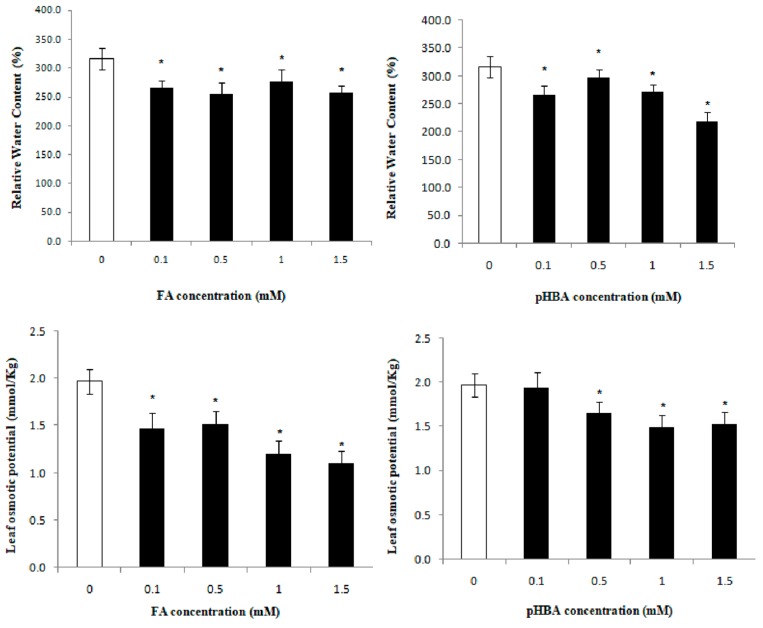
Leaf relative water content (%) and leaf osmotic potential (mmol/Kg) in leaves of *Rumex acetosa* L. one week after exposure to four concentrations (0.1, 0.5, 1.0, and 1.5 mM) of ferulic acid (FA) and *p*-hydroxybenzoic acid (pHBA) and control. Every column in each graph represents the mean (± S.E.) of three replicates. * Asterisks indicate significant differences at *p* ≤ 0.05 with respect to control.

Exposure of the *R. acetosa* seedlings at 0.1–1.5 mM FA corresponded to a reduction in RWC from 15.18% to 18.48% (Figure 2). Following pHBA treatment, RWC significantly decreased (30.9%) at 1.5 mM as compared to control. The leaf osmotic potential (LOP) was significantly decreased after FA and pHBA treatments. FA reduced the LOP (45%) in *R. acetosa* at highest concentration tested (Figure 2). pHBA significantly reduced the LOP in a gradual manner and this inhibition was 23.5%, as compared to control (Figure 2). This demonstrate that FA is more toxic and significantly decreased the LOP in *R. acetosa*.

### 3.4. Unrevealing the Inhibitory Effects of Phenolic Acids in Planta

The phenolic compound (FA and pHBA) impact on *R. acetosa* was very destructive at all levels. The leaf fresh weight (LFW) was decreased by 53.5% and 48.5% following treatment at 0.1 and 1.5 mM FA (Table 2), respectively. The pHBA decreased the LFW and more severe damage was observed at 1.5 mM where reduction was 43.8% compared to control. In *R. acetosa*, the leaf dry weight was decreased by 66.6% and 57.5% after treatment with FA and pHBA at 1.5 mM level (Table 2), respectively. The root fresh weight (RFW) of *R. acetosa* was significantly decreased at all concentrations of phenolic acid and the maximum reduction in RFW was obtained following treatment at 1.5 mM FA (62.9%) and pHBA (52.4%), as compared to control. Root dry weight (RDW) was decreased by 60.2% and 48.9% after treatment with FA and pHBA, respectively, compared to control (Table 2). Shoot length (SL) in *R. acetosa* was decreased after treatment with 1.5 mM concentration of FA and pHBA, by 22.78% and 21.67%, respectively, as compared to the control. The results revealed that pHBA (1.5mM) can suppress root length of *R. acetosa* by up to 33.47% compared to the control (Table 2). The *R. acetosa* seedlings treated with 1.5 mM FA had similar patterns of variation, and caused significant reduction in RL (24.4%).

## 4. Discussion

The yield of major cereal and food crops significantly declines due to the heavy infestation of different weeds in the agriculture fields. Furthermore, several weed flora have developed resistance against herbicides due to the continuous application of the same compound in a particular crop species and on the same piece of land. Some of the broad leaf weeds are the major cause of yield reduction in Pakistan and *Rumex dentatus* is just an example that brought a threshold decline in the wheat yield [23]. Natural products such as secondary metabolites from plants, fungi and bacteria can be used for the safe development of lead compounds for new bioherbicides discovery program [24].

Secondary metabolites have been demonstrated to obstruct several growth, ecophysiological and biochemical aspects of crops, weeds and horticultural species. Several researchers have documented that secondary metabolites have phytotoxicity and interfere with growth and ecophysiological functions such as photosynthesis, respiration, water status, and gene expression [6,25]. The field application of sorghum crop residue (which possesses several secondary metabolites, e.g., benzoic acid, p-hydroxybenozoic acid, vanillic acid, m-coumaric acid, p-coumaric acid, gallic acid, caffeic acid, ferulic acid and chlorogenic acid), incorporated into the soil, suppressed 20–48% of the growth and biomass of several weed species (*Chenopodium album*, *Phalaris minor*, *Avena fatua, Rumex dentatus*, *Senebiera didyma*, *Polygonum bellardi* and *Anagalis arvensis*). The sorghum residue was more lethal because it decreased the density of *Cyperus rotundus* by 28–92% [26]. Phytotoxic and inhibitory effects of phenolic compounds, e.g., ferulic acid, *p*-hydroxybenzoic acid, and cinnamic acid, produced by certain plants on the germination, growth and ecophysiology traits of other crops have been reported [15,17,25,26]. Several hundred secondary metabolites were previously identified from different sources but few of them have been evaluated for their biological activity against crops and weeds [7]. Microorganisms are also a substantial source of secondary metabolites. Some authors have recently isolated, and identified 14 allelochemicals from microbial source and elucidated their phytotoxic mode of action [27].

Plant photosynthesis discriminates against the stable ^13^C isotope, when atmospheric CO_2_ passes through stomata and during CO_2_ carboxylation in RuBisCO [20]. The variations in stable isotopes of C and N contain a potential wealth of information regarding phenotypic plasticity because stable isotope ratios are reliable indicators of spatially and temporally dynamic changes in environment. Leaf Δ^13^C was decreased in *R. acetosa* plants grown with decreasing RWC (Figure 1 and Figure 2). Similar results have been reported for wheat [19], barley [28], Russian wild rye [29], *Lolium perenne* [17], *Lactuca sativa* [14] and rice [30]. In the present study, allelochemical stress caused difficulties to the plant for extracting the water as indicated by the gradual decrease in RWC. Similar results were documented for rice genotypes by Zhao et al. [30], and alfalfa by Erice et al. [31]. Changes in concentration of FA and pHBA also led to changes iWUE and leaf Δ^13^C. These findings are in accordance with the theory published by Farquhar and Richards [19] that the relationship between wheat WUE and plant biomass may be positive or negative [32]. In a study conducted on sunflower by Virgona and Farquhar [33], a positive correlation was observed between the studied traits (WUE vs biomass).

Barkosky and Einhellig [34] demonstrated that δ^13^C values in soybean plants were less discriminated following treatment with pHBA. It was observed that a reduction in intercellular CO_2_ concentration might be due to closed stomata that led to reduced rate of WUE and subsequent higher Δ^13^C. Similarly, Δ^13^C values in lettuce leaves were lower than control following treatment with BOA [14]. The Δ^13^C values significant reduced because the *C*i/*C*a process is inhibited at elevated level of both phenolic acids. This coincided with unbalance supply of CO_2_ from inside to outside of stomata [17]. There was significant decrease in *C*i/*C*a values following increase in N_leaf_ that might stimulate photosynthesis and decrease in *C*i/*C*a through RuBisCO stimulation. It may also increase transfer towards photosynthetic organs (chloroplasts). 

Agro-physiological attributes of *R. acetosa* (LFW, LDW, SL, and RL) were reduced following treatment with both secondary metabolites. RFW and RL of *R. acetosa* were decreased after pHBA at 0.5–1.0 mM treatment. Similarly, agro-morphological traits, especially root growth in pea (*Pisum sativum* L.), were decreased following treatment with *p*-coumaric and *p*-hydroxybenzoic acids [35]. Secondary metabolites are reported to inhibit root growth and the modification of root morphology and histology [36]. In a field study, Anjum and Bajwa [37] evaluated sunflower aqueous extracts and synthetic herbicides (Buctril-Super and Chwastox) on *Rumex dentatus* in wheat field plots. They found that sunflower aqueous extracts reduced the growth and biomass of broad leaf weeds. Several authors have reported the high specific inhibitory nature of synthetic molecules (herbicides) but this is not true for secondary metabolites that often possess multi-site action in plants and it is difficult to separate the primary and secondary impact of natural compounds. Consequently, these features prohibit the wide application and their practical application for weed management programs in the field conditions. As compared to commercial herbicides, secondary metabolites also possess several modes of action and inhibit physiological, biochemical, growth, and isotopic processes [6]. The herbicide properties of a secondary metabolite, dehydrozaluzanin C, are significantly better than Logran herbicide and their efficacy is more prominent on broad leaf weeds [38]. 

Both allelochemicals caused significant reduction in C_leaf_ and N_leaf_ concentration. Similar results were reported by Hirel et al. [39] who found a similar decreasing trend in C_leaf_ and N_leaf_ concentration after abiotic stress. It has been observed that allelochemicals released from some donor plants into the environment might interfere the absorption and availability of certain nutrients to other plants. The soil environment can be modified through root exudates and litters that affect soil structure, biochemical properties, and nutrient mobilization. Li et al. [40] demonstrated that the growth of maize intercropped with *Vicia faba* was improved due to availability of phosphorus. They found that organic acids released from the root exudates helps to increase the phosphorus supply to the maize plant. Our results indicate that there was 25–29% reduction in leaf δ^15^N in *R. acetosa*. According to the literature, several abiotic stress factors (salinity, drought, and allelochemicals) can either decrease [41,42] or increase δ^15^N [43]. Evans [44] documented that N isotopes can provide integrated information about nitrogen fluxes, assimilation pathways and allocation.

However, the lack of physiological, biochemical and genomic techniques and bioassays are the major constraints in the practical application of allelopathy in weed management programs in field settings. Due to the advancement in the scientific research, many new equipment and protocols have been added by several researchers and scientists belonging to agronomy, plant physiology, plant stress physiology, molecular biology and biophysics that have significantly removed the hurdles and made the results more reliable [4,6,7,11,17,18]. It has been demonstrated that rye plant releases several phenolic compounds into the surrounding soils that can arrest the growth and development of obnoxious weed, *Avena fatua* L. [18]. The same author successfully recovered 5 kg ha^−1^ of secondary metabolite (benzoxazinones) from soil leachates following rye decomposition. However, the complete pathway for several natural products remains unclear. Therefore, it is important that further scientific investigation open new avenues for better perception of modern approaches to exploit crop allelopathy in organic weed management [45]. 

## 5. Conclusions 

Secondary metabolites (FA and pHBA) significantly affected the seedling growth, and physiological and biochemical features of adult plants of broad leaf weed *Rumex acetosa*. Allelochemical effects were modulated through shoot and root growth inhibition, altering leaf water contents and leaf osmotic potential traits. Allelochemical treatments led to the inhibition of biochemical attributes of stable isotopes of C and N. The present study clearly demonstrates that leaf water relation combined with carbon isotope composition traits are reliable indicators of more stable, accurate and suitable methodologies in allelopathic research. It also highlighted the impedance in δ^13^C and *C*i/*C*a, providing significant insights into the plant ecophysiological and biochemical attributes, which led to better understanding of the fundamental aspects of plant stress physiology. Further study under field conditions are scheduled to find the cause of the environmental toxicity, and the interaction of these compounds with soil particles and microorganisms, as well as their movement and degradation consequences.

## Figures and Tables

**Table 1:** Effect of different concentrations of ferulic acid (FA) and p-hydroxybenzoic acid (pHBA) on carbon and nitrogen concentrations and nitrogen isotope composition (δ^15^N) of *Rumex acetosa*.

Treatments	Concentration (mM)	C%	N%	C/N ratio	δ^15^N
	Control	48.14 ± 1.9 a	1.97 ± 0.31 a	24.43 ± 0.6 b	15.02 ± 0.4 a
FA	0.1	46.02 ± 5.5 b	1.90 ± 0.39 a	24.22 ± 0.4 b	11.09 ± 3.5 b
	0.5	46.37 ± 1.9 b	1.52 ±0.4 c	30.5 ± 0.4 a	10.46 ± 1.1 c
	1	43.66 ± 2.5 c	1.82 ±0.63 b	23.98 ± 0.5 c	11.59 ± 0.9 b
	1.5	44.28 ± 1.2 c	1.47 ± 0.32 c	30.12 ± 0.6 a	10.77 ± 0.9 c
	Control	48.14 ± 1.9 a	1.97 ± 0.31 a	24.43 ± 0.6 c	15.02 ± 0.4 a
pHBA	0.1	44.88 ± 3.5 b	1.95 ± 0.68 a	23.01 ± 0.43 c	11.56 ± 1.3 b
	0.5	43.96 ± 0.4 c	1.65 ± 0.36 b	26.64 ± 0.6 b	10.21 ± 0.7 c
	1	43.34 ± 4.2 c	1.53 ± 0.38 c	28.32 ± 0.4 a	10.58 ± 1.3 c
	1.5	43.61 ± 3.2 c	1.40 ± 0.40 d	31.15 ± 0.5 a	9.94 ± 1.0 d

C%, foliage carbon concentration; N%, foliage nitrogen concentration; C/N ratio, ratio of carbon to nitrogen; δ^15^N, stable nitrogen isotope composition. The values are the means (± S.E.) of three replicates per treatment. Means followed by different letters are significantly different (*p* ≤ 0.05).

**Table 2:** Effect of ferulic acid (FA) and p-hydroxybenzoic acid (pHBA) at different concentrations (0, 0.1, 0.5, 1.0, and 1.5 mM) on agro-morphological traits of *Rumex acetosa* L.

Treatments	Concentration (mM)	LFW	LDW	RFW	RDW	SL	RL
Control	Control	2.39 a	0.33 a	3.05 a	0.98 a	15.32 a	19.18 a
FA	0.1	1.11 c	0.27 b	1.99 c	0.633 b	11.83 c	16 b
	0.5	1.01 c	0.21 b	1.13 c	0.71 b	12.16 b	14.33 c
	1	0.99 c	0.12b c	2.62 b	0.816 b	10.56 c	17 b
	1.5	1.23 b	0.11 b	3.11 a	0.396 c	12.73 b	14.5 c
pHBA	0.1	0.55 c	0.09 c	2.12 a	0.793 a	12 b	14.83 b
	0.5	0.77 b	0.16 b	2.01 a	0.646 b	12.5 b	14.33 b
	1	0.93 a	0.18 a	1.78 b	0.606 b	12.26 b	19 a
	1.5	0.98 a	0.14 c	1.6 b	0.506 c	13a	12.76 c

Each value represents the mean (± S.E.) of three replicates. Means (*n* = 3) with different letters indicate significant difference at *p* ≤ 0.05. Leaf fresh weight, LFW; leaf dry weight, LDW; root fresh weight, RFW; root dry weight, RDW; shoot length, SL; root length, RL.

## Abbreviations

iWUE	intrinsic water use efficiency
LOP	leaf osmotic potential
RWC	relative water content
δ^13^C	composition of carbon isotope ratios
Δ^13^C	carbon isotope discrimination
*C*i/*C*a	ratio of intercellular CO_2_ concentration from leaf to air
